# Complete mitochondrial genome of *Cheilio inermis* (Labriformes, Labridae)

**DOI:** 10.1080/23802359.2018.1501319

**Published:** 2018-08-17

**Authors:** Ha Yeun Song, Seonmi Jo, Seung-Hyun Jung, Hyun-Ju Hwang, Hye Suck An

**Affiliations:** Department of Genetic Resources Research, National Marine Biodiversity Institute of Korea, Seocheon-gun, Republic of Korea

**Keywords:** *Cheilio inermis*, Labriformes, Labridae, mitochondrial genome

## Abstract

In the present report, we describe the first sequencing and assembly of the complete mitochondrial genome of *Cheilio inermis*. The mitochondrial genome of *C*. *inermis*, with 16,494 bp in length, has the typical vertebrate mitochondrial gene arrangement. It contains 13 protein-coding genes, 22 tRNA genes, two rRNA genes, and a control region. All the tRNA genes typically formed a cloverleaf secondary structure. Phylogenetic analysis using mitochondrial genomes of 11 species showed that *C. inermis* formed monophyletic group with other Labridae species.

The cigar wrasse, *Cheilio inermis* (Labriformes: Labridae), is widely distributed throughout the tropical/subtropical Indo-Pacific region, from the Red Sea and East Africa to the Hawaiian and Easter Islands (Randall et al. 1990; Cheung et al. 2010). This fish inhabits seagrass beds and algal-covered flats, occasionally in lagoon and seaward reefs (Myers 1991; Kuiter and Tonozuka 2001; Gell and Whittington 2002) and is assessed as Least Concern in IUCN Red List (Cheung et al. 2010). To our knowledge, this study is the first to determine the sequence of complete mitochondrial genome of *C. inermis*, and to analyse the phylogenetic relationship of this species with members of Labridae.

The *C. inermis* specimen used in this study was collected from Chuuk ST 1, Micronesia (7.27N, 151.54E). Total genomic DNA was isolated from tissue of the specimen, which has been deposited in the National Marine Biodiversity Institute of Korea (Voucher No. MABIK 0000619). The mitogenome was sequenced and assembled using Illumina Hiseq 4000 sequencing platform (Illumina, San Diego, CA) and SOAPdenovo assembler at Macrogen Inc. (Seoul, Korea), respectively. The complete mitochondrial genome was annotated using MacClade ver. 4.08 (Maddison and Maddison 2005) and DNASIS ver 3.2 (Hitachi Software Engineering, Tokyo, Japan). 

The complete mitochondrial genome of *C. inermis*, with 16,494 bp in length (GenBank accession no. AP018552), and includes 13 protein-coding genes, 22 tRNA genes, two rRNA genes, and a control region (D-Loop). The *ND6* gene and eight tRNA genes are encoded on the light strand. The overall base composition of the heavy strand is 26.49% A, 30.26% C, 17.42% G, and 25.83% T. Similar to the mitogenomes of other vertebrates, the purine-pyrimidine base pairing of AT content is higher than the GC content (Saccone et al. 1999). All tRNA genes can fold into a typical cloverleaf structure, with lengths ranging from 65 to 75 bp. The 12S rRNA (948 bp) and 16S rRNA genes (1,693 bp) are located between tRNA^Phe^ and tRNA^Val^ and between tRNA^Val^ and tRNA^Leu(UUR)^, respectively. Of the 13 protein-coding genes, 12 begin with an ATG start codon; the exception being the *COI* gene, which start with GTG. For the stop codon of the protein-coding genes, five genes ended with a single base T, *ATP6* and *COIII* with TA, *ND6* with TAG and *NDI*, *COI*, *ATP8*, *ND4L* and *ND5* with TAA. The control region is 827 bp long and located between tRNA^Pro^ and tRNA^Phe^.

Phylogenetic trees were constructed by the maximum-likelihood method using MEGA 7.0 software (Kumar et al. 2016) for the newly sequenced genome and a further 10 complete mitochondrial genome sequences downloaded from the National Center for Biotechnology Information. We confirmed that *C. inermis* formed a monophyletic group with other Labridae species with high statistical support (Figure 1). The newly determined mitochondrial genome in this study will provide important DNA molecular data for further phylogenetic analysis and conservation genetics of *C. inermis*.

**Figure 1.**
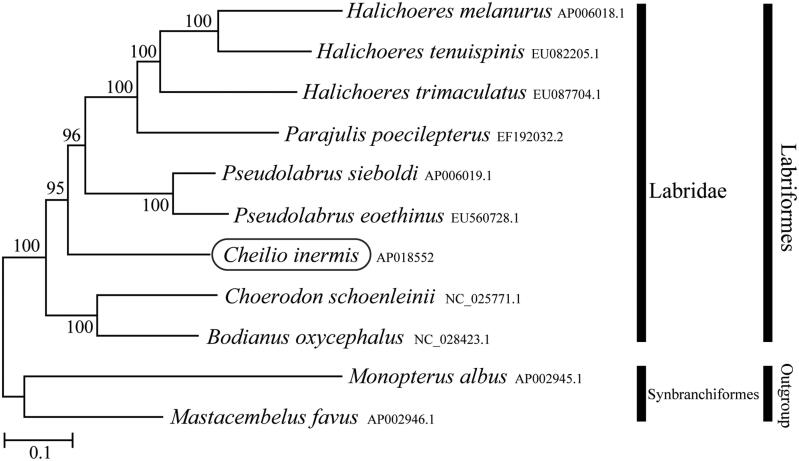
Phylogenetic position of *Cheilio inermis* based on a comparison with the complete mitochondrial genome sequences of 10 species. The analysis was performed using MEGA 7.0 software. The accession number for each species is indicated after the scientific name.
